# Supplementary material for: Fatty acids homeostasis during fasting predicts protection from chemotherapy toxicity
Source: Nat Commun. 2022 Sep 27;13:5677. doi: 10.1038/s41467-022-33352-3 (PMC9515185; doi:10.1038/s41467-022-33352-3)
Supplement: Supplementary file 1 — Supplementary Info [file 41467_2022_33352_MOESM1_ESM.pdf]

Fatty acids homeostasis during fasting predicts protection from chemotherapy toxicity.

Inventory of Supporting Information

Marta Barradas[1,13,*], Adrián Plaza[1,13,*], Gonzalo Colmenarejo[2], Iolanda Lázaro[3], Luis Filipe Costa-Machado[1], Roberto Martín-Hernández[2], Victor Micó[4], José Luis López-Aceituno[1], Jesús Herranz[2], Cristina Pantoja[1], Hector Tejero[5], Alberto Diaz-Ruiz[6], Fatima Al-Shahrour[5], Lidia Daimiel[4], Viviana Loria-Kohen[7], Ana Ramirez de Molina[7,8], Alejo Efeyan[9], Manuel Serrano[10], Oscar J. Pozo[11], Aleix Sala-Vila[3,12], Pablo J. Fernandez-Marcos[1,*].

- Supplementary Figure S1.
- Supplementary Figure S2.
- Supplementary Figure S3.
- Supplementary Figure S4.
- Supplementary Figure S5.
- Supplementary Figure S6.
- Supplementary Figure S7.
- Supplementary Table 1.

Figure S1

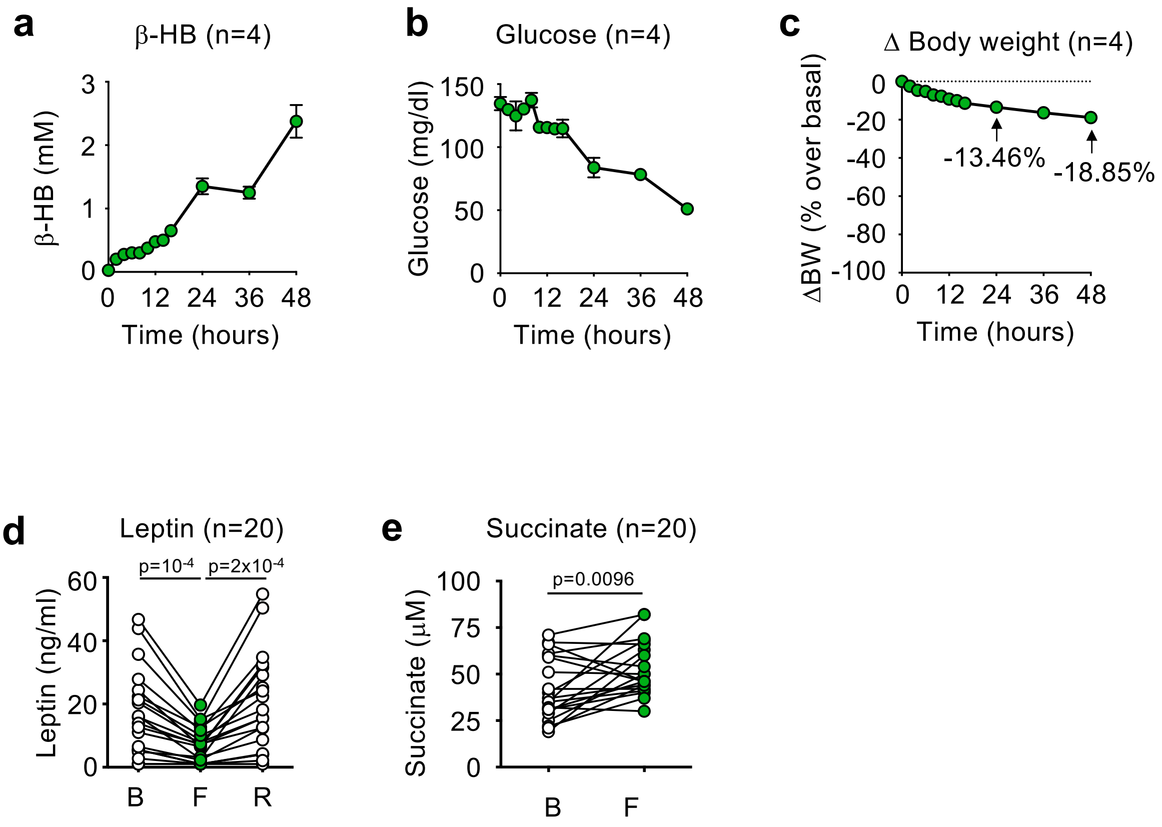

Supplementary Figure S1. Biochemical and morphometric markers in fasting mice and humans. (a-c) 12-week-old male mice were put to fast and the blood ketone β-hydroxybutyrate (β-HB, a), blood glucose (b) and the change in body weight (ΔBW, expressed as a % of basal weight) compared to the initial weight (c) were recorded at the indicated times. **(d-e)** Blood levels at the indicated times of leptin (d) or succinate (e) in the human volunteers shown in Figure 1 at the basal point after 12 hours of overnight fasting (B); after 36 hours of fasting (F); and after 12 hours of refeeding and 12 hours of overnight fasting (R). Statistical significance was assessed using the one-way ANOVA with Tukey correction for multiple comparisons (d) or two-tailed paired Student t test (e). The exact p value is provided for each significant comparison. Source data are provided as a Source Data file.

Figure S2

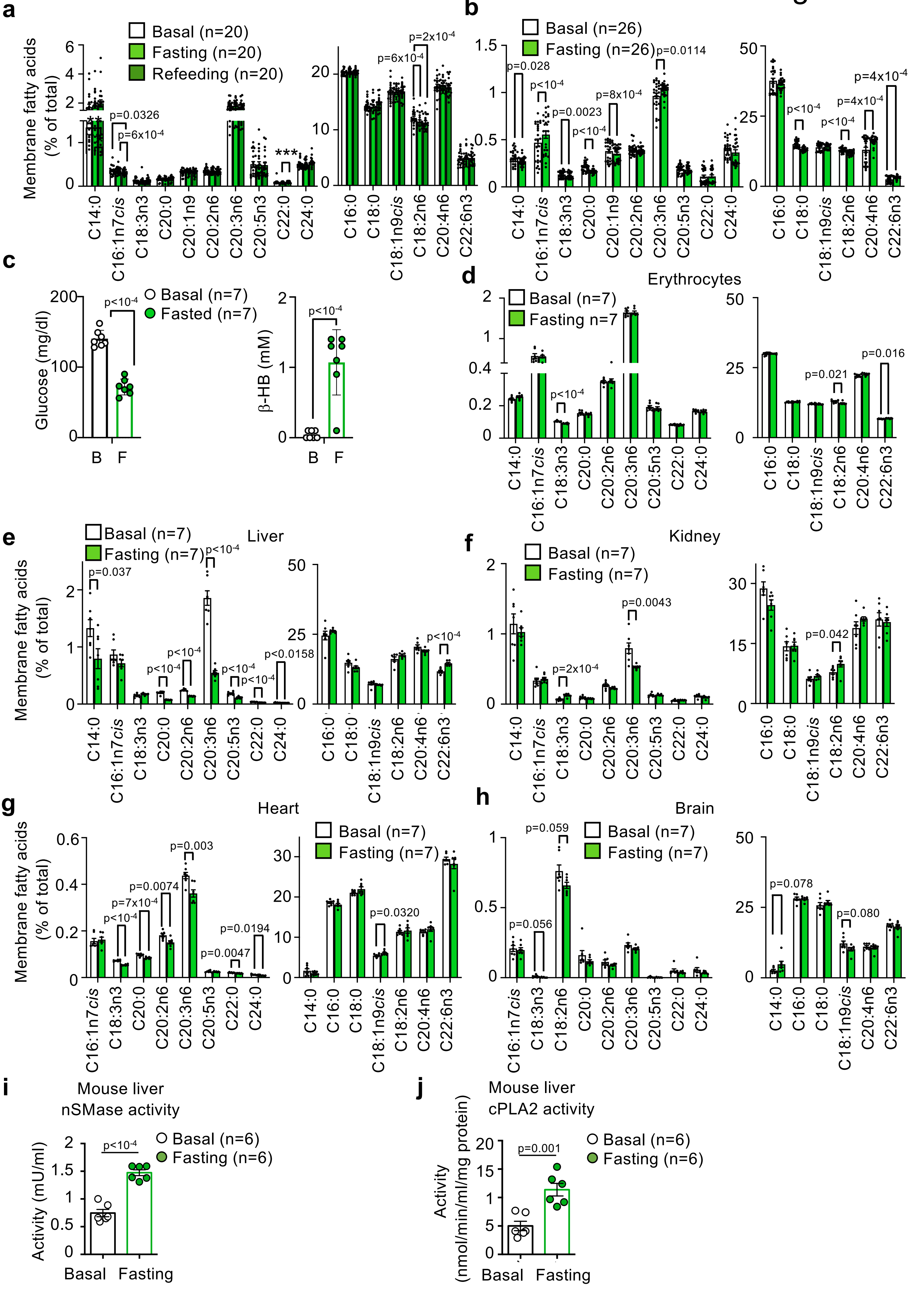

a
Basal (n=20)
Fasting (n=20)
Refeeding (n=20)
Membrane fatty acids (% of total)
p=0.0326
p=6x10-4
***
p=6x10-4
p=2x10-4
b
Basal (n=26)
Fasting (n=26)
p=0.028
p<10-4
p=0.0023
p<10-4
p=8x10-4
p=0.0114
p<10-4
p<10-4
p=4x10-4
p=4x10-4
c
Basal (n=7)
Fasted (n=7)
Glucose (mg/dl)
β-HB (mM)
p<10-4
B
F
d
Erythrocytes
Basal (n=7)
Fasting n=7
p<10-4
p=0.021
p=0.016
e
Liver
Basal (n=7)
Fasting (n=7)
p=0.037
p<10-4
p<0.0158
f
Kidney
Basal (n=7)
Fasting (n=7)
p=0.0043
p=2x10-4
p=0.042
g
Heart
Basal (n=7)
Fasting (n=7)
p=0.003
p=0.0074
p=7x10-4
p<10-4
p=0.0047
p=0.0194
p=0.0320
h
Brain
Basal (n=7)
Fasting (n=7)
p=0.059
p=0.056
p=0.078
p=0.080
C14:0
C16:1n7cis
C18:3n3
C20:0
C20:1n9
C20:2n6
C20:3n6
C20:5n3
C22:0
C24:0
C16:0
C18:0
C18:1n9cis
C18:2n6
C20:4n6
C22:6n3
i
Mouse liver
nSMase activity
Activity (mU/ml)
p<10-4
Basal (n=6)
Fasting (n=6)
Basal
Fasting
j
Mouse liver
cPLA2 activity
Activity (nmol/min/ml/mg protein)
p=0.001
Basal (n=6)
Fasting (n=6)
Basal
Fasting

Supplementary Figure S2. Membrane fatty acids during fasting in human erythrocytes and mouse tissues. (**a-b**) Quantification of the % of erythrocyte membrane fatty acids in the human (a) and mouse (b) cohorts shown in Figures 1 and 2 at the indicated time points: Basal, after 12 hours of fasting (humans, a) or in fed condition (mice, b); Fasting, after 36 hours (humans, a) or 24 hours (mice, b) of fasting; Refeeding: after 12 hours of refeeding and 12 hours of fasting (humans, a). **(c-h**) Two groups of 12-week-old male mice were either kept under normal feeding (Fed) or fasted for 24 hours (Fasted), and then sacrified to obtain blood and tissue samples. Correct fasting was verified by measuring blood glucose and β-HB (c). The indicated membrane fatty acids from cell membranes of erythrocytes (d), liver (e), kidney (f), heart (g) and brain (h) were measured. **(i, j)** Activities of the neutral sphingomyelinase (nSMase, i) or the cytosolic phospholipase A2 (cPLA2, j) in livers from mice fed normally (Fed) or fasted for 24 hours (Fasting). Bars represent the average of the indicated number of individuals. Error bars represent the standard error of the mean. Statistical significance was assessed using one-way ANOVA with Tukey correction for multiple comparisons (a), and the paired (b) or unpaired (c-j) two-tailed Student t test. The exact p value is provided for significant comparisons. Source data are provided as a Source Data file.

Figure S3

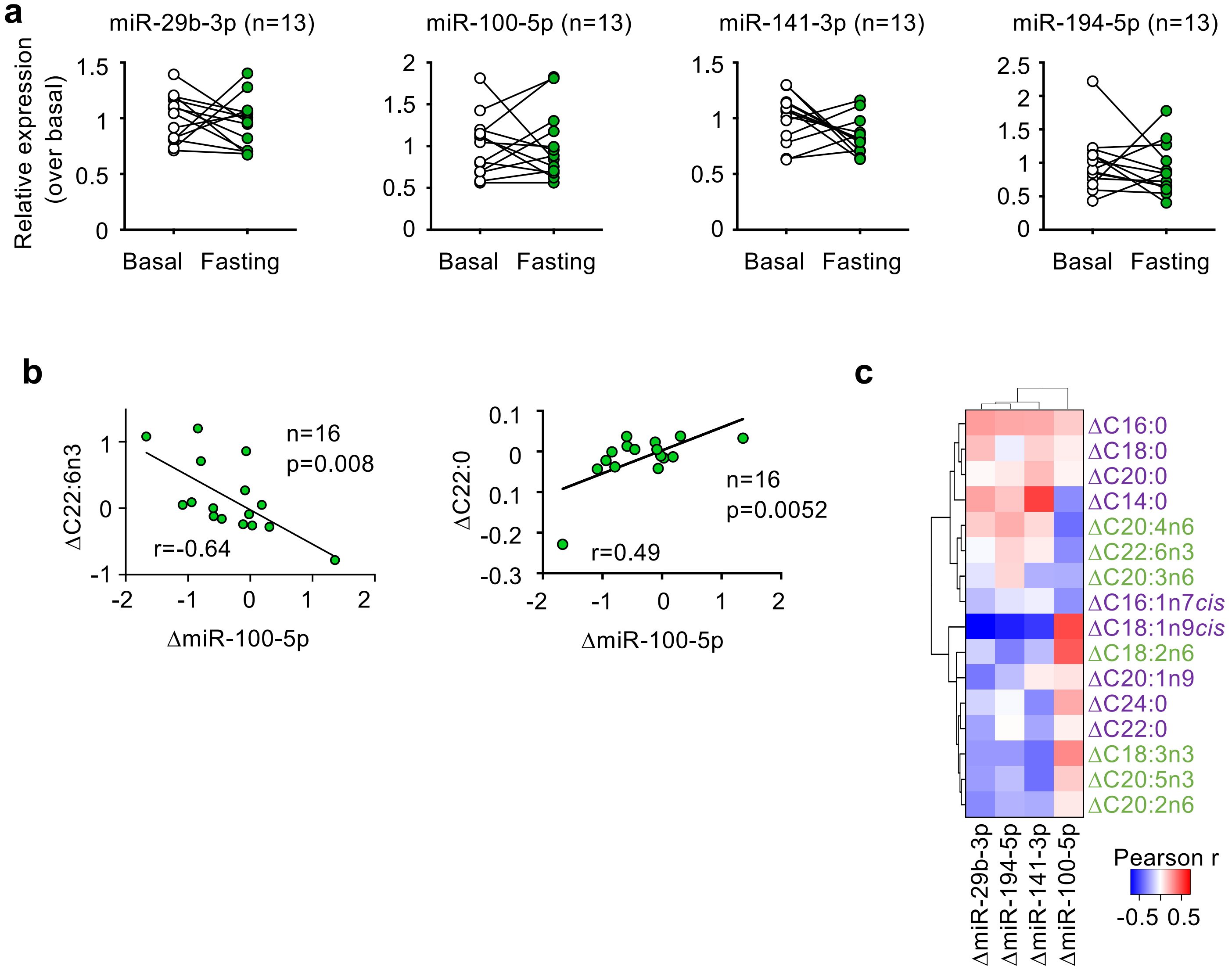

Supplementary Figure S3. Circulating miRNA changes during short-term fasting. (a) Expression of the indicated miRNAs measured in plasma from mice at the basal point and after a 24 hour-fasting. **(b)** Linear correlation of the changes with fasting (fasting *minus* basal) of the indicated parameters measured in human plasma samples. **(c)** Heatmap showing the Pearson correlation r values between changes (fasting minus basal) of all the analyzed erythrocyte plasma membrane fatty acids and changes with fasting of the indicated circulating miRNAs significantly altered with fasting in mice. Each dot represents data from one precise individual. Lines in (a) link the paired observations from the same individual. Statistical significance was calculated using the two-tailed paired Student t test (a) or the Pearson correlation (b). The exact p value is provided for significant comparisons. Source data are provided as a Source Data file.

Figure S4

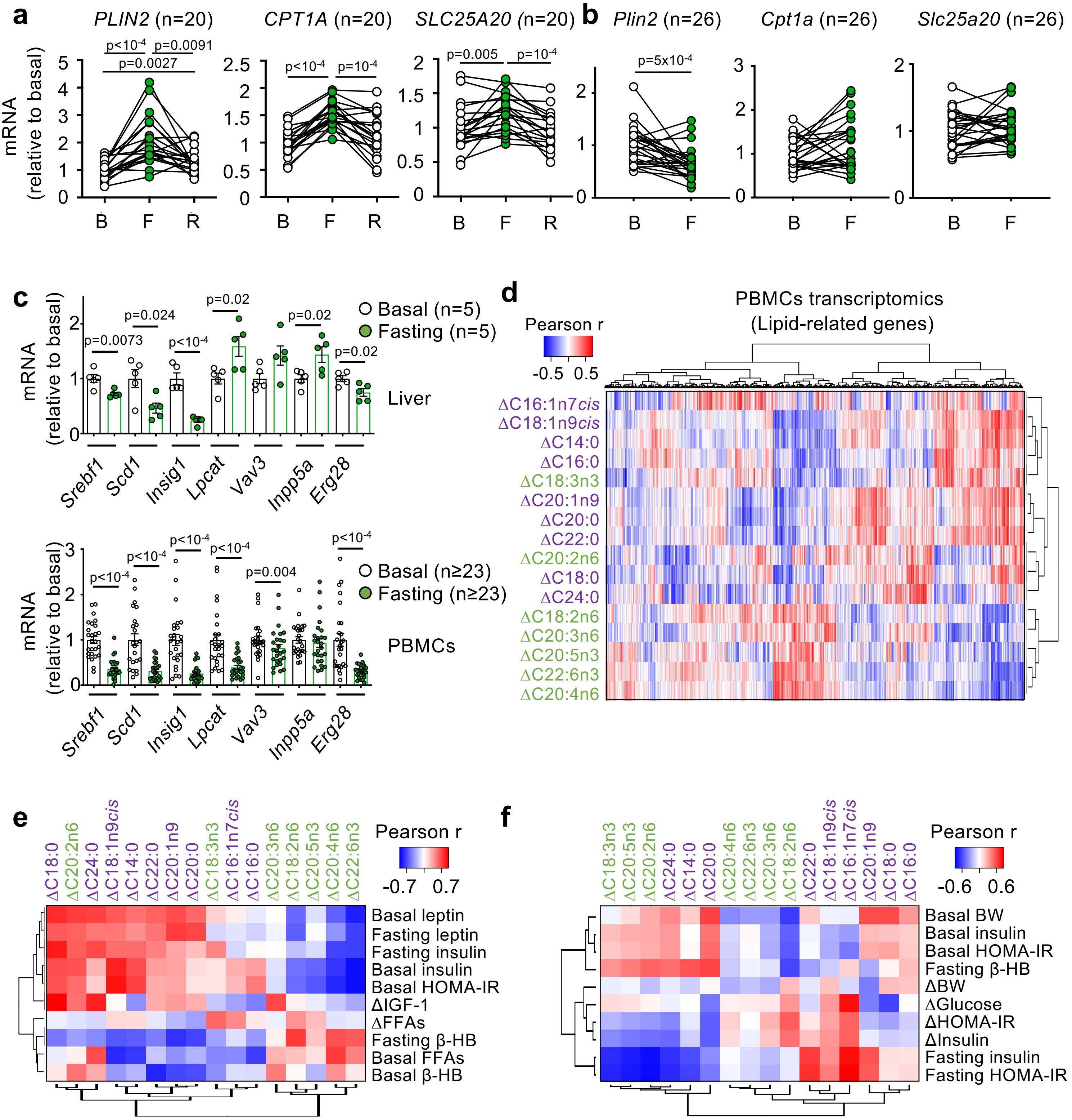

Supplementary Figure S4. Changes in gene expression during fasting in humans and mice. (a, b) Gene expression at PBMCs from humans (a) or mice (b) at the indicated times of the indicated PPARα target genes. **(c)** mRNA expression of the indicated genes in livers (top panel) or PBMCs (bottom panel) of mice on normal feeding (Basal) or fasted for 24 hours (Fasting). **(d)** Heatmap depicting the correlation of changes with fasting of all lipid-related genes detected in the RNAseq from human peripheral blood mononuclear cells (a total of 923 genes) and the changes with fasting of the indicated erythrocyte membrane fatty acids (SFAs and MUFAs are written in purple and PUFAs are written in green). **(e, f)** Pearson correlations of changes in erythrocyte membrane fatty acids with the most significant biochemical and morphometric parameters shown in Table 3 for humans (e) and mice (f). HOMA-IR: Homeostatic Model Assessment for Insulin Resistance. FFAS: free fatty acids. β-HB: β-hydroxybutyrate. BW: body weight. Line-connected dots represent the paired data for each individual (a and b), while bars represent the average of the indicated replicates (c). Error bars represent the standard error of the mean. Statistical significance was assayed using the one-way ANOVA with Tukey correction for multiple comparisons (a); the unpaired (c, top panel) or paired (b, c-bottom panel) two-tailed Student t test; or Pearson correlations (d-f). The exact p value is provided for significant comparisons. Source data are provided as a Source Data file.

Figure S5

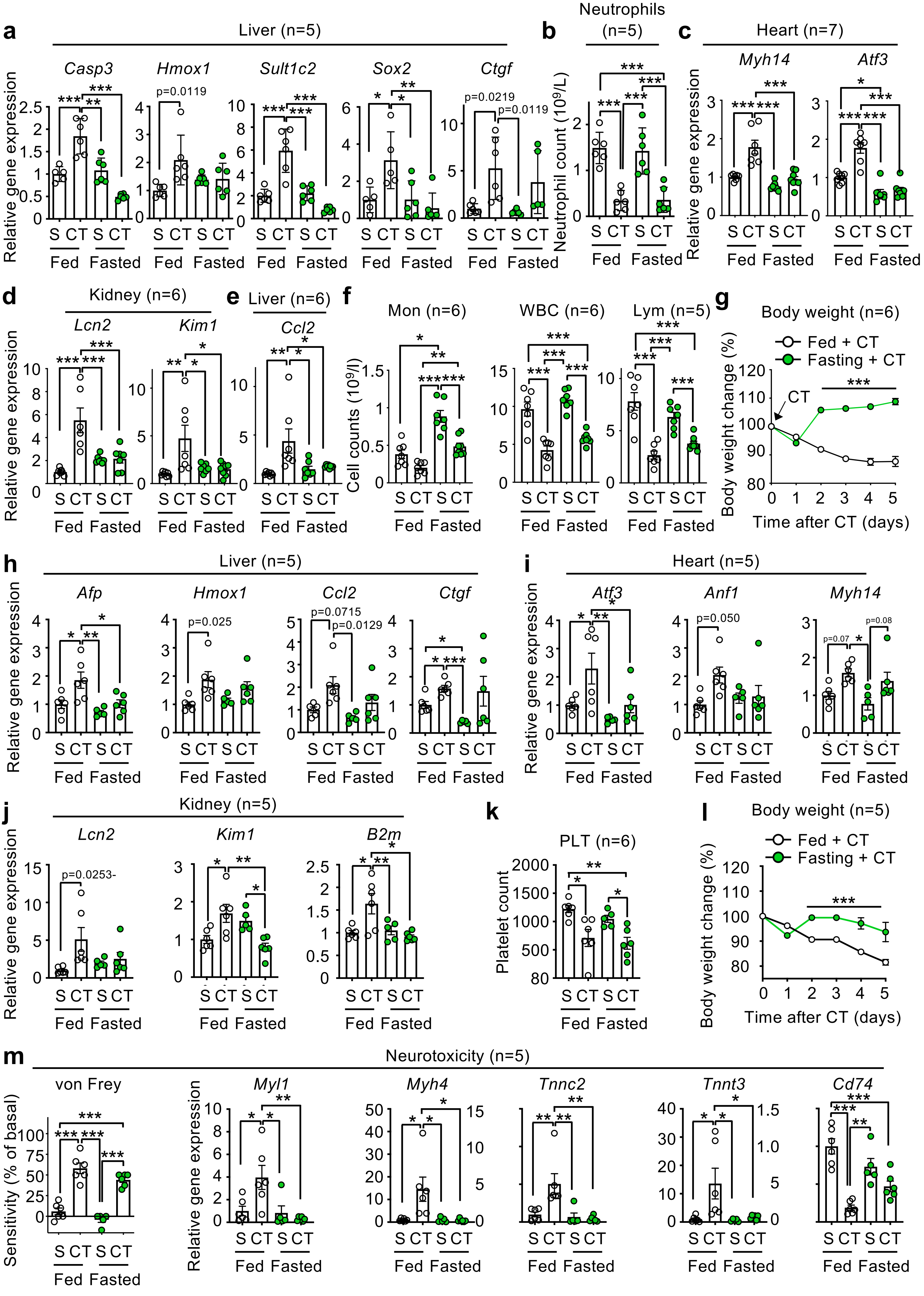

a
Liver (n=5)
Casp3
Hmox1
Sult1c2
Sox2
Ctgf
p=0.0119
p=0.0219
p=0.0119
Relative gene expression
S CT S CT
Fed Fasted
b
Neutrophils (n=5)
Neutrophil count (10^9/L)
c
Heart (n=7)
Myh14
Atf3
d
Kidney (n=6)
Lcn2
Kim1
e
Liver (n=6)
Ccl2
f
Mon (n=6)
WBC (n=6)
Lym (n=5)
Cell counts (10^9/l)
g
Body weight (n=6)
Fed + CT
Fasting + CT
CT
Body weight change (%)
Time after CT (days)
h
Liver (n=5)
Afp
Hmox1
Ccl2
Ctgf
p=0.025
p=0.0715
p=0.0129
i
Heart (n=5)
Atf3
Anf1
Myh14
p=0.050
p=0.07
p=0.08
j
Kidney (n=5)
Lcn2
Kim1
B2m
p=0.0253-
k
PLT (n=6)
Platelet count
l
Body weight (n=5)
Fed + CT
Fasting + CT
Body weight change (%)
Time after CT (days)
m
Neurotoxicity (n=5)
von Frey
Sensitivity (% of basal)
Myl1
Myh4
Tnnc2
Tnnt3
Cd74
Relative gene expression
S CT S CT
Fed Fasted

Supplementary Figure S5. Different fasting protocols protect from different chemotherapy toxicities. (a, b) Expression of the indicated toxicity-reporter genes in liver (a) or blood neutrophils counts (b) from mice shown in Figure 5 either fed ad libitum or fasted for 24 hours before and 24 hours after i.p. inoculation mice of saline (S) or 15 mg/kg oxaliplatin (CT), and sacrificed 5 days after chemotherapy administration. **(c-g)** Expression of the indicated toxicity-reporter genes in heart (c), kidney (d) or liver (e); counts of the indicated lymphocyte populations (f; Mon: monocytes; WBC: white blood cells; Lym: lymphocytes); and body weight (g) at the indicated time points after chemotherapy (CT) administration from mice either fed ad libitum or fasted for 24 hours before and 24 hours after i.p. inoculation of saline (S) or 15 mg/kg doxorubicin (CT). **(h-m**) Expression of the indicated toxicity-reporter genes in liver (h), heart (i) or kidney (j); blood platelet counts (PLT, k); body weight (l); and neurotoxicity biomarkers (m) including the scores for the neurotoxicity-measuring experiment von Frey test (m, panel to the left) and expression of the indicated toxicity genes in dorsal root ganglia (m, panels to the right) from mice either fed ad libitum or fasted for 12 hours before and 12 hours after i.p. inoculation of saline (S) or 15 mg/kg oxaliplatin (CT), and sacrificed 5 days after chemotherapy administration. Bars and line-connected dots represent the average of $n \geq 5$ mice per treatment group. Error bars represent the standard error of the mean. Statistical significance was assayed using the one-way ANOVA test (a-f, h-k, m) or the repeated measures two-way ANOVA test (g, l) with Tukey correction for multiple comparisons. When possible, the exact p value is provided for significant comparisons; otherwise, *, $p<0.05$, **, $p<0.01$; ***, $p<0.001$. Source data are provided as a Source Data file.

Figure S6

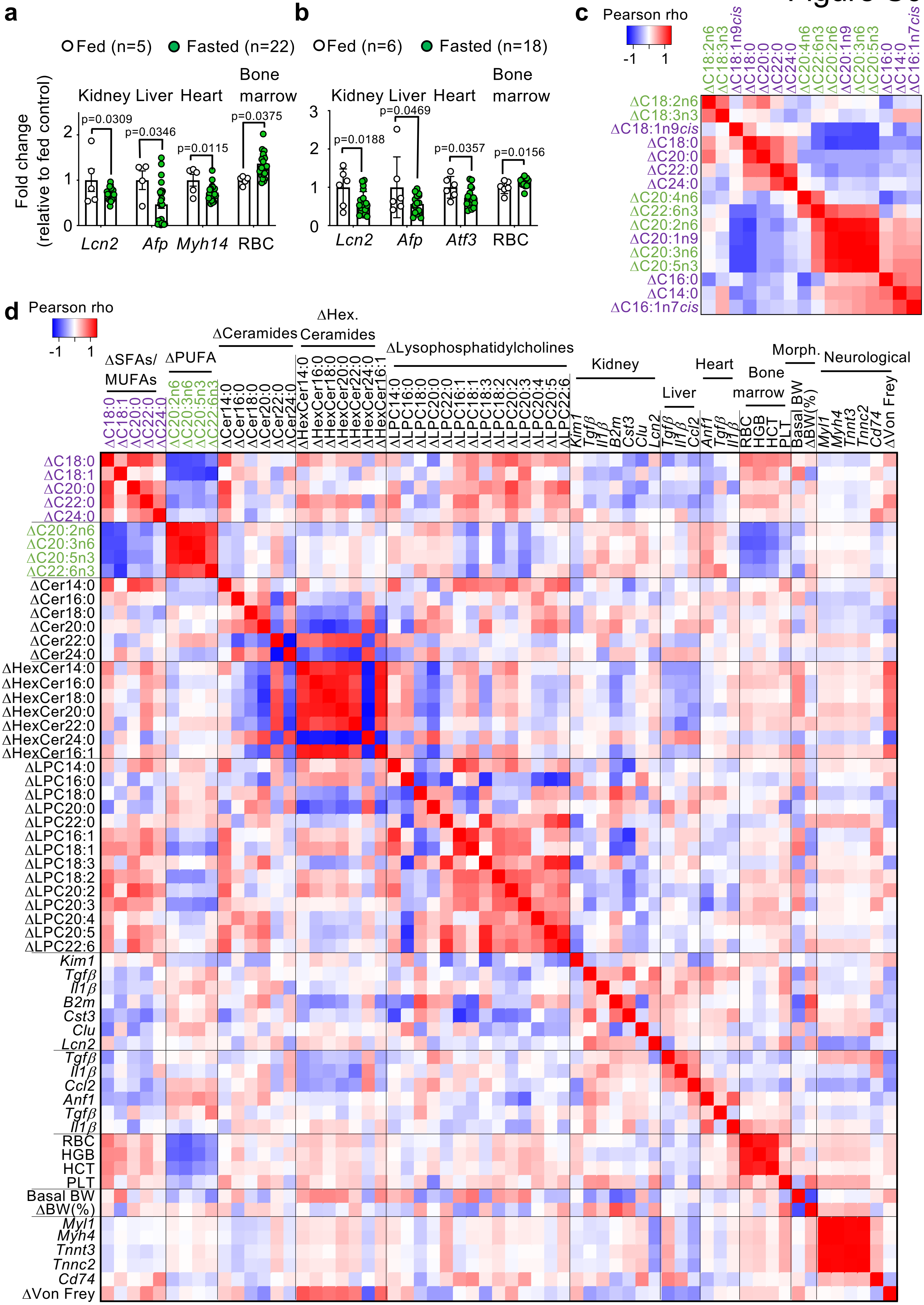
a
Fed (n=5)
Fasted (n=22)
Fold change (relative to fed control)
Kidney
Liver
Heart
Bone marrow
p=0.0309
p=0.0346
p=0.0115
p=0.0375
Lcn2
Afp
Myh14
RBC
b
Fed (n=6)
Fasted (n=18)
p=0.0188
p=0.0469
p=0.0357
p=0.0156
Lcn2
Afp
Atf3
RBC
c
Pearson rho
-1
1
ΔC18:2n6
ΔC18:3n3
ΔC18:1n9cis
ΔC18:0
ΔC20:0
ΔC22:0
ΔC24:0
ΔC20:4n6
ΔC22:6n3
ΔC20:2n6
ΔC20:1n9
ΔC20:3n6
ΔC20:5n3
ΔC16:0
ΔC14:0
ΔC16:1n7cis
d
Pearson rho
ΔSFAs/ MUFAs
ΔPUFA
ΔCeramides
ΔHex. Ceramides
ΔLysophosphatidylcholines
Kidney
Liver
Heart
Bone marrow
Morph.
Neurological
ΔC18:0
ΔC18:1
ΔC20:0
ΔC22:0
ΔC24:0
ΔC20:2n6
ΔC20:3n6
ΔC20:5n3
ΔC22:6n3
ΔCer14:0
ΔCer16:0
ΔCer18:0
ΔCer20:0
ΔCer22:0
ΔCer24:0
ΔHexCer14:0
ΔHexCer16:0
ΔHexCer18:0
ΔHexCer20:0
ΔHexCer22:0
ΔHexCer24:0
ΔHexCer16:1
ΔLPC14:0
ΔLPC16:0
ΔLPC18:0
ΔLPC20:0
ΔLPC22:0
ΔLPC16:1
ΔLPC18:1
ΔLPC18:3
ΔLPC18:2
ΔLPC20:2
ΔLPC20:3
ΔLPC20:4
ΔLPC20:5
ΔLPC22:6
Kim1
Tgfβ
Il1β
B2m
Cst3
Clu
Lcn2
Tgfβ
Il1β
Ccl2
Anf1
Tgfβ
Il1β
RBC
HGB
HCT
PLT
Basal BW
ΔBW(%)
Myl1
Myh4
Tnnt3
Tnnc2
Cd74
ΔVon Frey

Supplementary Figure S6. Lipidomics of mouse erythrocyte membranes and its association with chemotherapy toxicity. (a) Expression of the indicated toxicity biomarker genes in the indicated tissues, in the mice shown in Figure 6, subjected to 15 mg/kg oxaliplatin inoculation and either fed ad libitum (Basal) or fasted for 24 hours before and 24 hours after (Fasting) oxaliplatin administration. **(b)** Expression of the indicated toxicity biomarker genes in the indicated tissues, in a new cohort of mice subjected to the same treatment as in (a). **(c)** Heatmap representing the Pearson correlation coefficients (r) between the changes with fasting of erythrocyte membrane fatty acids of the mice shown in (b) after the first 24 hours of fasting. **(d)** Heatmap representing the Pearson correlation coefficients (r) between the changes with 24 hours of fasting (Δ) of the indicated lipids carrying one single fatty acid (total fatty acids, ceramides, hexosyl-ceramides and lysophosphatidylcholines), and the indicated toxicity biomarkers obtained at sacrifice. RBC: red blood cells. HGB: hemoglobin. HCT: hematocrit. PLT: platelets. BW: body weight. Bars represent the average of the indicated number of mice per treatment group. Error bars represent the standard error of the mean. Statistical significance was assayed using the unpaired two-tailed Student t test. The exact p value is provided for significant comparisons. Source data are provided as a Source Data file.

Figure S7

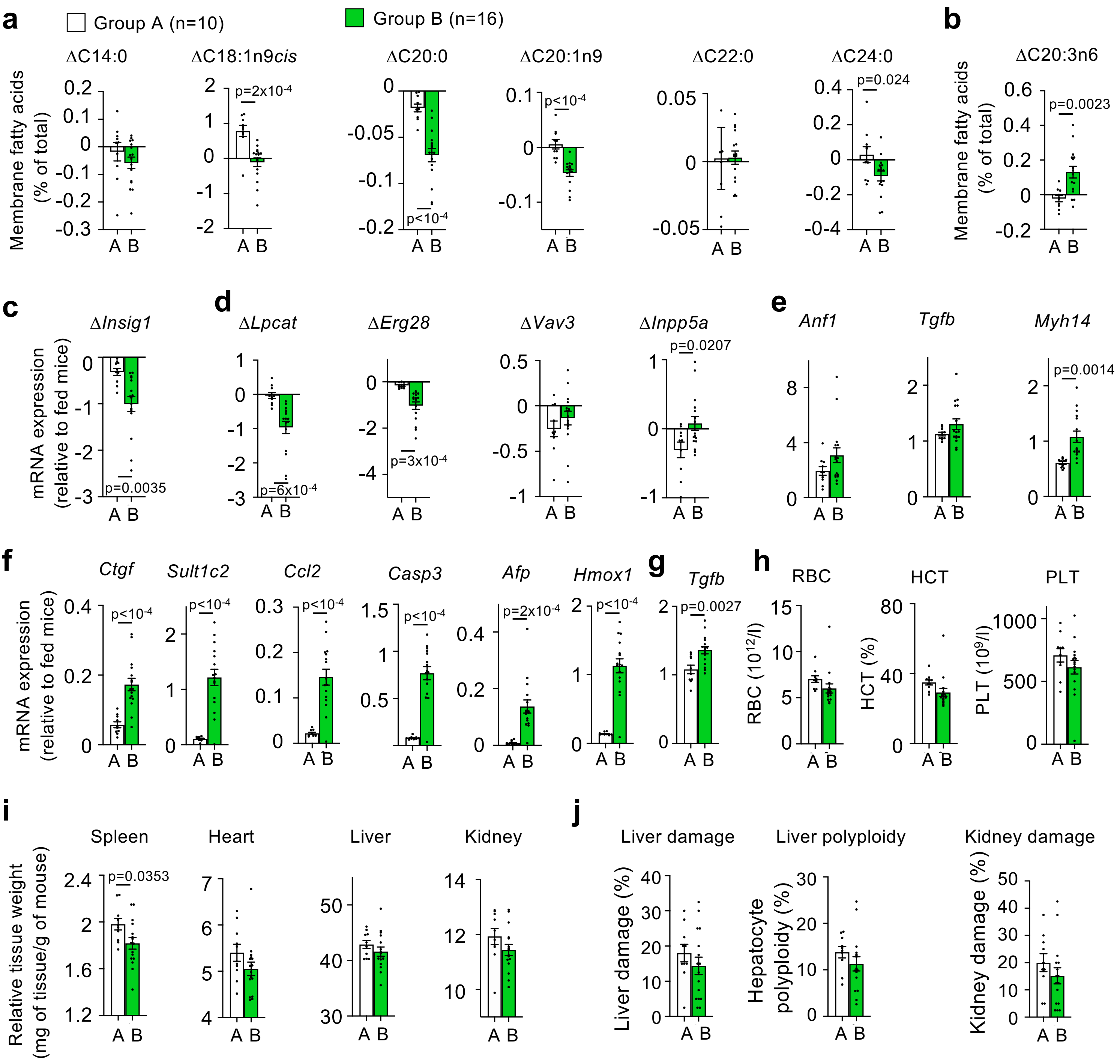

Supplementary Figure S7. Distribution of biomarkers of fasting and chemotherapy toxicity in mice according to the fasting-response groups. (a-j) Groups A and B, identified in Figures 4f and 7a, were compared for the indicated parameters: changes with fasting of the levels of the indicated SFAs and MUFAs (a) or PUFAs (b) in the erythrocyte membranes; changes with fasting of the expression of the indicated genes in PBMCs (c, d); expression of the indicated toxicity genes in heart (e), liver (f) and kidney (g) 5 days after chemotherapy administration; hematological parameters (h; RBC: red blood cells; HCT: hematocrit; PLT: platelets); relative weights of the indicated tissues (i); and histological findings as described in Figure 5 (j). Bars represent the mean of n=10 (group A) and n=16 (group B). Error bars represent the standard error of the mean. Statistical significance was assessed using the unpaired two-tailed Student t test. The exact p value is provided for significant comparisons. Source data are provided as a Source Data file.

Supplementary Table 1

Human
**Parameter**
Age
Height
Basal body weight
BMI
Total fat %
Lean mass %
Visceral fat classification
Basal Metabolism
Waist
Systolic pressure
Diastolic pressure
Heart rate
Basal glucose
Fasting glucose
ΔGlucose
Basal insulin
Fasting insulin
ΔInsulin
Basal HOMA-IR
Fasting HOMA-IR
ΔHOMA-IR
Basal leptin
Fasting leptin
ΔLeptin
Basal β-HB
Fasting β-HB
Δβ-HB
Basal FFAs
Fasting FFAs
ΔFFAs
Basal IGF1
Fasting IGF1
ΔIGF1

Mouse
**Parameter**
Basal body weight
ΔBody weight
Basal glucose
Fasting glucose
ΔGlucose
Basal insulin
Fasting insulin
ΔInsulin
Basal HOMA-IR
Fasting HOMA-IR
DHOMA-IR
Fasting β-HB

Supplementary Table 1: list of the morphometric, physiological and biochemical parameters used to generate Table 3
