# Supplementary material for: Fatty acids homeostasis during fasting predicts protection from chemotherapy toxicity
Source: Nat Commun. 2022 Sep 27;13:5677. doi: 10.1038/s41467-022-33352-3 (PMC9515185; doi:10.1038/s41467-022-33352-3)
Supplement: Supplementary file 3 — Reporting Summary [file 41467_2022_33352_MOESM3_ESM.pdf]

nature portfolio

Corresponding author(s): Pablo J. Fernandez-Marcos, Marta Barradas, Adrián Plaza

Last updated by author(s): 3-8-2022

Reporting Summary

Nature Portfolio wishes to improve the reproducibility of the work that we publish. This form provides structure for consistency and transparency in reporting. For further information on Nature Portfolio policies, see our Editorial Policies and the Editorial Policy Checklist.

Statistics

For all statistical analyses, confirm that the following items are present in the figure legend, table legend, main text, or Methods section.

n/a	Confirmed	
☐	☒	The exact sample size (*n*) for each experimental group/condition, given as a discrete number and unit of measurement
☐	☒	A statement on whether measurements were taken from distinct samples or whether the same sample was measured repeatedly
☐	☒	The statistical test(s) used AND whether they are one- or two-sided<br>*Only common tests should be described solely by name; describe more complex techniques in the Methods section.*
☐	☒	A description of all covariates tested
☐	☒	A description of any assumptions or corrections, such as tests of normality and adjustment for multiple comparisons
☐	☒	A full description of the statistical parameters including central tendency (e.g. means) or other basic estimates (e.g. regression coefficient) AND variation (e.g. standard deviation) or associated estimates of uncertainty (e.g. confidence intervals)
☐	☒	For null hypothesis testing, the test statistic (e.g. *F*, *t*, *r*) with confidence intervals, effect sizes, degrees of freedom and *P* value noted<br>*Give P values as exact values whenever suitable.*
☒	☐	For Bayesian analysis, information on the choice of priors and Markov chain Monte Carlo settings
☐	☒	For hierarchical and complex designs, identification of the appropriate level for tests and full reporting of outcomes
☐	☒	Estimates of effect sizes (e.g. Cohen's *d*, Pearson's *r*), indicating how they were calculated

Our web collection on statistics for biologists contains articles on many of the points above.

Software and code

Policy information about availability of computer code

Data collection	No software was used for data collection.
Data analysis	Data was analyzed using the R package, version 4.0.2., and Prism 8 for macOS, version 8.4.3.

For manuscripts utilizing custom algorithms or software that are central to the research but not yet described in published literature, software must be made available to editors and reviewers. We strongly encourage code deposition in a community repository (e.g. GitHub). See the Nature Portfolio guidelines for submitting code & software for further information.

Data

Policy information about availability of data

All manuscripts must include a data availability statement. This statement should provide the following information, where applicable:

- Accession codes, unique identifiers, or web links for publicly available datasets
- A description of any restrictions on data availability
- For clinical datasets or third party data, please ensure that the statement adheres to our policy

The datasets generated during and/or analysed during the current study are available in the Zenodo repository, 10.5281/zenodo.6958418 (human and mouse dataset); and in the GEO repository, GSE173241 (RNAseq from human PBMCs).

Human research participants

Policy information about studies involving human research participants and Sex and Gender in Research.

Reporting on sex and gender	Sex and gender terms were used clearly and appropriately throughout the manuscript. Findings in humans apply to both sexes, making sure that 50% of participants were males/females (10 males and 10 females). Sex/gender data is provided in a disaggregated way in Table 1 in the manuscript. Consent for sharing individual disaggregated data was obtained.
Population characteristics	50% female Caucasian participants, 19<BMI<30, 22-46 years of age.
Recruitment	Participants were recruited via the Cantoblanco Platform for Nutritional Genomics and Food “GENYAL”, promoted by IMDEA Food, in an unbiased fashion.
Ethics oversight	All human protocols were approved by the Research Ethics Committee of the Madrid Institute for Advanced Studies (IMDEA) Food Foundation (PI-0025), according to the standards of the Helsinki Declaration.

Note that full information on the approval of the study protocol must also be provided in the manuscript.

Field-specific reporting

Please select the one below that is the best fit for your research. If you are not sure, read the appropriate sections before making your selection.

- [x] Life sciences
- [ ] Behavioural & social sciences
- [ ] Ecological, evolutionary & environmental sciences

For a reference copy of the document with all sections, see nature.com/documents/nr-reporting-summary-flat.pdf

Life sciences study design

All studies must disclose on these points even when the disclosure is negative.

Sample size	Previous reports on similar human (Fazeli et al., 2016, with n=11 volunteers; Bouwens et al., 2007, with n=4 volunteers) or mouse (Lopez-Guadamillas et al., 2016) projects yielded statistically significant results with similar sample sizes. Four our mouse studies, we used a larger cohort to stratify individuals according to their response to fasting.
Data exclusions	No data were excluded from the analyses.
Replication	At least 3 independent replicates for each parameter were analyzed, and, where possible (Figure 5, replicated in Figure S5a-b; Figure 6, replicated in Figure S6), measurements and analyses were reproduced 2 or more times, and only findings that were consistent between repeats were included in the manuscript.
Randomization	In the experiments including groups, group allocation was random, insuring homogeneous age, sex and metabolic status for all groups before the interventions.
Blinding	For human studies, no blinding was necessary, because all participants followed the same intervention. For mouse experiments, histological analysis were performed in a blinded fashion. For other molecular analyses of human and mouse samples, blinding was not performed because all samples were treated equally and measurements were obtained in an automated, non-subjective fashion.

Reporting for specific materials, systems and methods

We require information from authors about some types of materials, experimental systems and methods used in many studies. Here, indicate whether each material, system or method listed is relevant to your study. If you are not sure if a list item applies to your research, read the appropriate section before selecting a response.

Materials & experimental systems

n/a	Involved in the study	
x		Antibodies
x		Eukaryotic cell lines
x		Palaeontology and archaeology
	x	Animals and other organisms
	x	Clinical data
x		Dual use research of concern

Methods

n/a	Involved in the study	
x		ChIP-seq
x		Flow cytometry
x		MRI-based neuroimaging

Animals and other research organisms

Policy information about studies involving animals; ARRIVE guidelines recommended for reporting animal research, and Sex and Gender in Research

Laboratory animals	Mouse (Mus musculus), C57BL/6OlaHsd, males, 12-14 week-old. Mice were housed at 22ºC and with 12 hours dark/light cycles (light cycles from 7 a.m. to 7 p.m.), in constant humidity kept at 40-60%.
Wild animals	The study did not involve wild animals.
Reporting on sex	Only male mice were used for this study.
Field-collected samples	The study did not involve field-collected samples.
Ethics oversight	Animal experimentation at the National Center of Biotechnology (CNB, Madrid) was performed according to protocols approved by the CNB-CSIC Ethics Committee for Research and Animal Welfare (PROEX 148/18).

Note that full information on the approval of the study protocol must also be provided in the manuscript.

Clinical data

Policy information about clinical studies

All manuscripts should comply with the ICMJEguidelines for publication of clinical research and a completedCONSORT checklist must be included with all submissions.

Clinical trial registration	ClinicalTrials.gov Identifier: NCT04259879
Study protocol	https://clinicaltrials.gov/ct2/show/NCT04259879?term=imdea+food&draw=2&rank=4
Data collection	Data and samples were collected at the GENYAL platform, in the IMDEA Food institute headquarters, in June 2016.
Outcomes	Primary outcomes included gene expression measurements in PBMCs: Expression analysis of different genes from PBMCs were performed in a HT-7900 Fast Real time polymerase chain reaction (PCR). Quantifications were made applying the ΔCt method (ΔCt = [Ct of gene of interest - Ct of housekeeping]). The housekeeping genes used for input normalization were β-actin (ACTB) and ribosomal protein lateral stalk subunit P0 (RPLP0). Secondary outcomes included blood hormones and metabolites measurements (insulin, free fatty acids, ketone bodies, leptin, lipid profiles), using standardized protocols. Also, for a subjective evaluation of tolerance to fasting, participants filled in a fasting tolerance test based on the symptoms they feel, resulting in a final score of tolerance to fasting.
